# Age distribution and associated factors of cornea biomechanical parameter stress-strain index in Chinese healthy population

**DOI:** 10.1186/s12886-020-01704-6

**Published:** 2020-11-03

**Authors:** Guihua Liu, Hua Rong, Ruxia Pei, Bei Du, Nan Jin, Di Wang, Chengcheng Jin, Ruihua Wei

**Affiliations:** 1 Tianjin International Joint Research and Development Centre of Ophthalmology and Vision Science, Postal address: 251 Fukang Road, Nankai District, Tianjin, China; 2 Eye Institute and School of Optometry, Tianjin Medical University Eye Hospital, Postal address: 251 Fukang Road, Nankai District, Tianjin, China

**Keywords:** Corvis ST, Corneal biomechanics, Anterior segment parameters, Stress-strain index, Chinese population

## Abstract

**Background:**

To investigate the new cornea biomechanical parameter stress-strain index (SSI) in Chinese healthy people and the factors associated with SSI.

**Methods:**

A total of 175 eyes from 175 participants were included in this study. Axial length was measured with the Lenstar LS-900. Pentacam measured curvature of the cornea and anterior chamber volume (ACV). Cornea biomechanical properties assessments were performed by corneal visualization Scheimpflug technology (Corvis ST). Student’s t-test, one-way ANOVA, partial least square linear regression (PLSLR) and linear mixed effects (LME) model were used in the statistical analysis.

**Results:**

The mean (±SD) SSI was 1.14 ± 0.22 (range, 0.66–1.78) in all subjects and affected by age significantly after age of 35 (*P* < 0.05). In LME models, SSI was significantly associated with age (β = 0.526, *P* < 0.001), axial length (AL) (β = − 0.541, *P* < 0.001), intraocular pressure (IOP) (β = 0.326, *P* < 0.001) and steepest radius of anterior corneal curvature (RsF) (β = 0.229, *P* < 0.001) but not with ACV, biomechanical corrected intraocular pressure (bIOP), flattest radius of anterior corneal curvature (RfF) or central corneal thickness (CCT) (*P* > 0.05 for each).

**Conclusions:**

SSI increased with age after the age of 35. In addition to age, SSI was positively correlated with RsF and IOP, while negatively correlated with AL.

## Background

Corneal biomechanical determination is of great importance in clinical evaluation, such as diagnosis of keratoconus [1], assessment before corneal refractive surgery [2, 3], and measurement of corrected intraocular pressure [4, 5]. In recent years, various instruments that measured corneal biomechanics were developed, such as ocular response analyzer (ORA) and Corvis ST. Meanwhile, a variety of parameters to assess corneal biomechanics were provided. However, since cornea consists of a viscoelastic material, and its stress-strain behavior of biological tissue is nonlinear [6, 7], the cornea shows the biomechanical properties of changes under different intraocular pressure load. Hon reported a corneal indentation device (CID) that can measure the stiffness (S) and tangent modulus (E) of the cornea in vivo, but S and E due to IOP rise could not be ruled out [8, 9]. It was always a difficult problem to evaluate corneal biomechanics without the influence of IOP in vivo [10–12].

A new method to obtain the stiffness of corneal material was proposed by Eliasy, which eliminated central corneal thickness (CCT) and biomechanical corrected intraocular pressure (bIOP) in vivo [10]. A biomechanical Corvis parameter, stress-strain index (SSI) has been proved to be almost independent of bIOP and CCT. It was shown as an ideal method to estimate the mechanical properties of corneal tissue material. The distribution of SSI in Italian and Brazilian populations has been reported by previous studies and SSI was found to be significantly affected by age [10]. Chua observed that there was a lower corneal hysteresis (CH) in Chinese than Indians, suggesting that corneal biomechanics may be varies among different populations [13]. At present, the SSI distribution of different age in Chinese population has not been reported.

Corneal stiffness is one of the important biomechanical parameters, which reflects the ability to resist stress deformation [14]. Previous studies have shown that corneal biomechanics could be affected by multiple factors. A larger anterior chamber volume (ACV) was associated with a lower maximum amplitude at the apex of highest concavity (DA) and a higher stiffness parameter (SP-A1) in primary angle closure (PAC) eyes [15]. In healthy people, corneal biomechanical parameters were significantly correlated with the corneal curvature, refractive error [16, 17] but not with ACV [18]. However, Lim found that corneal biomechanics was not associated with age, refraction error, but related to CCT, cornea-compensated intraocular pressure (IOPcc) and anterior curvature [19]. While other factors that may influence SSI besides age have not been reported. Therefore, the purpose of this study is to explore the distribution of SSI in different age in healthy Chinese population and investigate the factors associated with SSI via Corvis ST.

## Methods

### Subjects

This was a prospective study on healthy eye cornea biomechanics. A total of 175 patients aged 5 to 74 years were recruited between May and June 2020 in Tianjin Medical University Eye Hospital. Written informed consent was obtained from all enrolled participants or their parents or guardians where participants were children (under 16 years old). All examinations conducted in accordance with the tenets of the Declaration of Helsinki and approved by Tianjin Medical University Eye Hospital ethics committee.

All patients underwent a complete ophthalmic examination, including visual acuity, slit-lamp ophthalmic examination, intraocular pressure, and fundus examination. Exclusion criteria included any corneal pathology, keratoconus, contact lens use, refractive surgery, uveitis, allergic eye disease, glaucoma or retinal disease, history of intraocular surgery, and any significant systemic illnesses. Patients with refractive error < −10D were excluded.

### Axial length

Axial length was measured with a non-contact biometer (Lenstar LS-900; Haag-Streit AG, Berne, Switzerland). Subjects were asked to keep both eyes open and fixate on the target. Between measurements, the subject was instructed to blink several times to make sure an intact tear film to prevent potential measurement errors. Three repeated measurements of the axial length were carried out and intra-session differences of no greater than 0.02 mm were averaged for data analysis.

### Curvature and ACV

Pentacam (Oculus, Wetzlar, Germany) was used to measure the curvature of cornea and ACV. All measurements were performed in a dark room. Patients were instructed to blink briskly before measurements taken and keep eyes widely open during scanning while fixating on the target. Only measurements which obtained an ‘OK’ quality index were saved.

### Biomechanical parameters

The cornea biomechanical properties were measured by Corvis-ST (Oculus, Wetzlar, Germany), which is a non-contact tonometer equipped with an optical pachymetry function. The corneal response to an air-puff pulse was recorded with 4330 images per second by a built-in high-speed camera. The following parameters were detected by Corvis ST: IOP, bIOP, CCT and the new parameter SSI. SSI is a parameter established to eliminate the interference of bIOP and corneal geometry and estimate the stiffness of the material, which is different from the stiffness parameter (SP). SSI algorithm was based on the prediction of cornea behavior by using the finite element numerical modeling simulation of the influence of IOP and Corvis ST air puff on cornea behavior. Only the quality index was ‘OK’ were included in the analysis.

### Statistical analysis

Statistical analyses were accomplished using SPSS statistical package 25 (SPSS, IBM, Chicago, IL, USA). Paired t-test was performed to explore the inter-ocular difference of SSI. There was no significant difference of SSI between the bilateral eyes (*P* > 0.05), thus, only left eyes were included in the subsequent analysis to avoid the bias of the relationship between bilateral eyes. Kolmogorov-Smirnov was used for testing the normal distribution of the data. The Student’s t-test was used to compare SSI between males and females. The sample population was divided into seven age groups by ten-year intervals ranging from 5 to 14 years to 65–74 years old. The differences among the subgroups according to age were compared using one-way ANOVA and the LSD. Because there were strong correlations between predictor variables, partial least square linear regression (PLSLR) was performed first, followed by linear mixed effects (LME) to reveal the relationship between SSI and clinical parameters. *P* < 0.05 was considered as statistically significant.

## Results

A total of 175 left eyes from 175 healthy patients who met the study inclusion criteria were analyzed. Among of them, 75 (42.9%) were male and 100 (57.1%) were female. SSI showed a normal distribution (*P* > 0.05). There were no significant gender differences existed in SSI (P > 0.05). The data are summarized in Table 1.

**Table 1:** Demographic and clinical characteristics of the study groups. *N* = 175

Parameters	Mean	SD	95%CI
SSI	1.14	0.22	1.11–1.17
Age, years	37.06	16.91	34.53–39.58
AL, mm	24.47	1.68	24.22–24.73
IOP, mmHg	14.91	1.62	14.67–15.15
bIOP, mmHg	14.36	1.56	14.13–14.59
RfF, mm	7.85	0.26	7.81–7.89
RsF, mm	7.62	0.26	7.58–7.66
ACV, mm^3^	167.75	44.07	161.18–174.33
CCT, μm	552.20	28.18	548.00–556.40

Data were expressed as mean ± standard deviation

*SSI* Stress-Strain index, *AL* Axial length, *IOP* Intraocular pressure, *bIOP* Biomechanical corrected intraocular pressure, *RfF* Flattest radius of anterior corneal curvature, *RsF* Steepest radius of anterior corneal curvature, *ACV* Anterior chamber volume, *CCT* Central corneal thickness, *SD* Standard deviation, *CI* Confidence interval

**Fig. 1:**
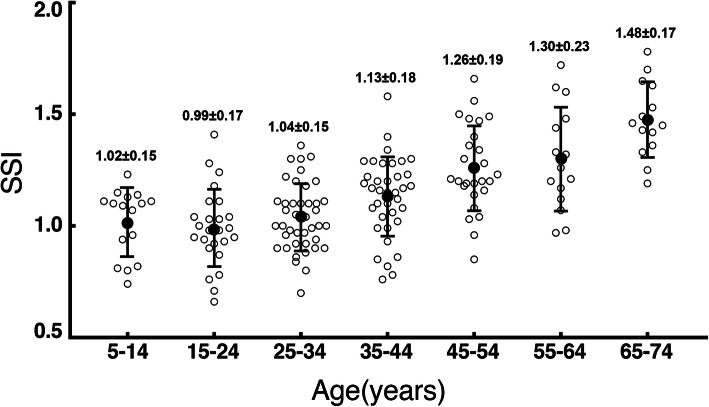
Mean values of the biomechanical parameters SSI for all subgroups according to age and shows the variation by age (ANOVA)

The average SSI was 1.14 ± 0.22 in all subjects and the values ranged from 0.66 to 1.78. To explore the differences of corneal stiffness between different age groups, patients were divided into seven groups according to age (Fig. 1). A significant rising trend was found in SSI with age increasing significantly after age of 35 (*P* < 0.05).

Due to the high correlation between the measurements, PLSLR and LME analysis was used to find the correlation between SSI and other parameters. After PLSLR calculation, five parameters were left, which were IOP, bIOP, AL, age and RsF, and then they were put into the LME model. The results showed that SSI was positively correlated with age, RsF and IOP, while it was negatively correlated with AL (Table 2).

**Table 2:** Coefficients (β) and *P*-value in LME models between each analysed variable and SSI

Parameters	LME model	
	β	***P***
Age, years	0.526	< 0.001^*^
AL, mm	−0.541	< 0.001^*^
IOP, mmHg	0.326	< 0.001^*^
RsF, mm	0.229	< 0.001^*^
bIOP, mmHg	0.070	0.445

*Statistically significant

*AL* Axial length, *IOP* Intraocular pressure, *RsF* Steepest radius of anterior corneal curvature, *bIOP* Biomechanical corrected intraocular pressure, *β* Standardized coefficients;

The adjusted R^2^ value of this LME model was 0.668. *P* < 0.05 considered statistically significant

## Discussion

This study described the distribution of corneal tissue material stiffness parameter SSI in different age groups and related factors in a healthy Chinese population. We found that SSI was relatively stable before age of 35, and then increased significantly with age. SSI was positively correlated with age, IOP, and anterior radius of curvature, meanwhile, it was negatively correlated with axial length. No significant effect was found in gender, ACV, CCT, or bIOP.

In our study, a nonlinear relationship was detected between age and SSI, showing that SSI increased with age significantly after age of 35. Age has been shown to be an important factor affecting corneal biomechanical in previous study [10]. Wang found a positive correlation between age and second applanation (A2L) in healthy Chinese adults [20]. In corneas of patients aged from 50 to 95 years, the tangent modulus increased with age [7]. Trend of corneal biomechanics with age could be accounted for changes in the molecular structure in cornea. Daxer and Malik observed that non-enzymatic crosslinking, collagen glycation, fibril diameter, and the number of collagen molecules increased with age over 40 years in corneal X-ray [21–23]. These could explain the reason that SSI increased with age after 35. However, we found that SSI was basically stable before the age of 35. In previous studies, Kirwan used ORA to measure corneas of normal children aged 4 to 18 years, and found that CH was not correlated with age [24]. Another study found that there was no significant correlation between biomechanical parameters and age in healthy Chinese adolescents at 4–18 years of age [25]. Valbon found that deformation amplitude (DA) and other biomechanical parameters of the healthy eyes in population under 40 years old were not correlated with age [26]. Elsheikh measured the corneal tangent modulus (E) in vitro for 30–99 years old, and found that the growth rate of E was smaller in the younger, which also suggested that the changes in corneal biomechanics may be uneven with age [27]. Unfortunately, few studies have reported the relationship between corneal collagen and age in young people. Whether this relationship is the reason that no significant correlation is revealed between SSI and age in the young remains to be explored. Furthermore, the AL and refractive error increase with age in young people [28]. We speculate those may reduce the tendency of the cornea to harden with age. This inference needs to be further proved in future studies.

SSI was found to be negatively correlated with axial length, which indicated that the SSI includes a function of the whole eye biomechanics and not just the cornea. Previous studies indicated that the cornea and sclera were mainly composed of the same types of collagen [29]. In addition, when the collagen fibers of the sclera became longer and damaged in myopia progression, the overall arrangement of collagen fibers in corneal stroma also restructured [30–32]. In infant monkeys and chicks, corneal astigmatism changes were also associated with induced eye growth [33, 34]. Chang’s study pointed out that the axial elongation led to corneal flattening and thickness reduction [35], suggesting that the increase of axial length may affect the biomechanics of the cornea and previous studies have proved this. Myopia in glaucoma and normal eyes would lead to biomechanical parameters changes such as corneal deformation amplitude (CDA), outward corneal applanation (OCA) and cornea stiffness (CS) [36, 37]. Especially the cornea in high myopia had faster outward corneal velocity (OCV) and higher CDA compared to emmetropia [16]. Long found in Chinese children, SP - A1 declined gradually between presbyopia, emmetropia and myopia groups [38]. However, in Lim’s study, CH and CRF were not significantly correlated with refractive errors by ORA measurement in corneal biomechanics in children aged 7–9 years. Lim believed the possible reason was the brief loading–unloading cycle of the ORA contrasts with the slower profile of scleral creep experiments and myopic deformation [19]. Actually, ORA exams CH or energy absorption but not corneal shape at maximum concavity. It may be more corneal specific and less surrounding affected and possibly buttressing sclera [14]. This may be the reason that biomechanical parameters measured by ORA are not correlated with refractive errors.

Consistent with the study of Eliasy, neither bIOP nor CCT was significantly correlated with SSI^10^, but SSI was found to be positively correlated with IOP. It was not surprising, since SSI reflected the corneal stiffness, and IOP measurement was affected by corneal stiffness [12, 39]. While it was emphasized that bIOP can exclude the influence of corneal thickness and age on intraocular pressure measurement [40], and can reflect more accurate intraocular pressure [41–43]. Besides IOP, previous studies have shown that, parameters of corneal biomechanics in vivo were mostly affected by bIOP such as deflection area at the highest concavity and deflection amplitude (HC DefArea and HC DefA), SP - A1 [43], CH and CRF [44]. However, SSI based on finite element (FE) numerical modeling simulats the effects of Corvis ST air puff and bIOP to predict of corneal behavior and excludes the influence of bIOP, indicating that this is an ideal method for in vivo measurement of corneal tissue material stiffness [10].

There was no significant correlation between SSI and ACV in LME model. The results of this study were consistent with Gabor Nemeth and Hwang, that the ACV was not significantly related to biomechanical parameters [18, 45]. Cui’s study pointed out that decrease in ACV led to reduced corneal stiffness in the PAC suspects. This could be explained by the different population in the studies. Cui believed that the stiffness of cornea would reduce in PAC and compensated for the high IOP caused by the shallow anterior chamber [15]. But in healthy people, there was no significant effect of ACV on corneal stiffness.

This study found that the radius of anterior curvature was positively correlated with SSI. There has been controversy on the relationship between corneal curvature and biomechanics. It was found that SP-A1 was positively correlated with corneal asphericity (Q value) and radius of anterior surface [46]. In Nemeth’s study, the corneal curvature was also related to the amplitude of corneal deformation and the time taken to reach this applanation [18]. On the contrary, several studies found that there was no significant correlation between the curvature and CH or CRF [45, 47, 48]. The correlation between corneal curvature and biomechanics needs further study.

Our research had several limitations. Firstly, SSI was currently estimated with normal corneal topography, which could not be applied in corneas with biomechanical decline caused by pathological changes. The calculation method of corneas with keratoconus or ectasia needs to be further developed. Secondly, the relationship between refractive error and SSI was not analyzed. Previous studies have pointed out that refractive error was highly correlated with the axial length of the eye [49], and it has also been shown that the extension of the axial length had a significant correlation with the sclera and cornea tissue structure [32]. Therefore, we considered that the axial length was more significant as an evaluation factor.

## Conclusions

In conclusion, this study for the first time conducted a statistical analysis of SSI in a healthy Chinese population and found that SSI was positively correlated with RsF, IOP and negatively correlated with AL. Moreover, after the age of 35, SSI increased with age.

## Data Availability

Data and materials are available upon request from the corresponding author at rwei@tmu.edu.cn.

## Abbreviations

Corvis ST: Corneal visualization Scheimpflug technology
AL: Axial length
IOP: Intraocular pressure
bIOP: Biomechanical corrected intraocular pressure
RfF: Flattest radius of anterior corneal curvature
RsF: Steepest radius of anterior corneal curvature
ACV: Anterior chamber volume
CCT: Central corneal thickness
